# Bis[4-chloro-2-(iminomethyl)phenolato]nickel(II)

**DOI:** 10.1107/S1600536809004279

**Published:** 2009-02-13

**Authors:** Zhe Hong

**Affiliations:** a College of Chemical Engineering and Materials Science, Liaodong University, Dandong 118003, People’s Republic of China

## Abstract

In the title centrosymmetric mononuclear nickel(II) complex, [Ni(C_7_H_5_ClNO)_2_], the Ni^II^ ion, lying on an inversion center, is four-coordinated by two O and two imine N atoms from two 4-chloro-2-imino­methyl­phenolate ligands, forming a distorted square-planar geometry. In the crystal structure, mol­ecules are linked into a two-dimensional network parallel to the *bc* plane by C—H⋯O hydrogen bonds.

## Related literature

For related structures, see: Hong (2007 ▶); Kamenar *et al.* (1990 ▶); Li *et al.* (2005 ▶, 2007 ▶); Zhou *et al.* (2004 ▶).
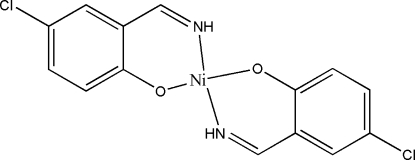


## Experimental

### 

#### Crystal data

[Ni(C_7_H_5_ClNO)_2_]
*M* *_r_* = 367.85
Monoclinic, 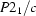

*a* = 15.775 (6) Å
*b* = 5.685 (2) Å
*c* = 7.894 (3) Å
β = 93.864 (18)°
*V* = 706.3 (5) Å^3^
*Z* = 2
Mo *K*α radiation
μ = 1.76 mm^−1^
*T* = 298 (2) K
0.18 × 0.17 × 0.17 mm

#### Data collection

Bruker SMART CCD area-detector diffractometer
Absorption correction: multi-scan (*SADABS*; Sheldrick, 1996 ▶) *T*_min_ = 0.743, *T*_max_ = 0.755
4102 measured reflections
1532 independent reflections
1296 reflections with *I* > 2σ(*I*)
*R*_int_ = 0.029

#### Refinement

*R*[*F* ^2^ > 2σ(*F* ^2^)] = 0.030
*wR*(*F* ^2^) = 0.084
*S* = 1.04
1532 reflections
100 parameters
1 restraint
H atoms treated by a mixture of independent and constrained refinement
Δρ_max_ = 0.45 e Å^−3^
Δρ_min_ = −0.30 e Å^−3^

### 

Data collection: *SMART* (Bruker, 2002 ▶); cell refinement: *SAINT* (Bruker, 2002 ▶); data reduction: *SAINT*; program(s) used to solve structure: *SHELXS97* (Sheldrick, 2008 ▶); program(s) used to refine structure: *SHELXL97* (Sheldrick, 2008 ▶); molecular graphics: *SHELXTL* (Sheldrick, 2008 ▶); software used to prepare material for publication: *SHELXTL*.

## Supplementary Material

Crystal structure: contains datablocks global, I. DOI: 10.1107/S1600536809004279/ci2768sup1.cif

Structure factors: contains datablocks I. DOI: 10.1107/S1600536809004279/ci2768Isup2.hkl

Additional supplementary materials:  crystallographic information; 3D view; checkCIF report

## Figures and Tables

**Table 1:** Hydrogen-bond geometry (Å, °)

*D*—H⋯*A*	*D*—H	H⋯*A*	*D*⋯*A*	*D*—H⋯*A*
C7—H7⋯O1^i^	0.93	2.54	3.318 (3)	142

Symmetry code: (i) 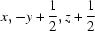.

**Fig. 1.**
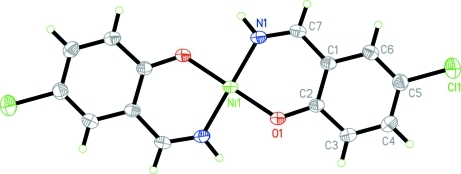
The molecular structure of the title compound, showing 30% probability displacement ellipsoids and the atom-numbering scheme. Unlabelled atoms are related to labelled atoms by the symmetry operation (1 - x, 1 - y, - z).

## supplementary crystallographic information

### Comment

Recently, the author has reported the crystal structure of a Schiff base nickel(II) complex (Hong, 2007). As an extension of the work on the structural investigation of nickel(II) complexes, the title complex is reported here.

The title compound is a centrosymmetric mononuclear nickel(II) complex (Fig. 1), which is similar to those reported previously (Kamenar *et al.*, 1990). The Ni atom, lying on the inversion center, is four-coordinated by two O and two imine N atoms from two 4-chloro-2-iminomethylphenol ligands, forming a square-planar geometry. The bond lengths (Table 1) related to the metal centre are comparable to the values in similar nickel(II) complexes (Zhou *et al.*, 2004; Li *et al.*, 2005; Li *et al.*, 2007).

In the crystal structure, the molecules are linked into a two-dimensional network parallel to the *bc* plane by C—H···O hydrogen bonds (Table 1).

### Experimental

5-Chlorosalicylaldehyde (1.0 mmol, 156.6 mg) and Ni(CH_3_COO)_2_.4H_2_O (0.5 mmol, 125.0 mg) were dissolved in methanol solution containing a small quantity of ammonia (30 ml). The mixture was stirred at room temperature for 30 min to give a clear brown solution. After keeping the solution in air for a few days, brown block-shaped crystals were formed.

### Refinement

Atom H1 was located from a difference Fourier map and refined isotropically, with the N1–H1 distance restrained to 0.90 (1) Å. Other H atoms were placed in idealized positions and constrained to ride on their parent atoms with C–H distances of 0.93 Å, and with *U*_iso_(H) set to 1.2*U*_eq_(C).

### Crystal data

**Table:**

[Ni(C_7_H_5_ClNO)_2_]	*F*(000) = 372
*M**_r_* = 367.85	*D*_x_ = 1.730 Mg m^−^^3^
Monoclinic, *P*2_1_/*c*	Mo *K*α radiation, λ = 0.71073 Å
Hall symbol: -P 2ybc	Cell parameters from 1954 reflections
*a* = 15.775 (6) Å	θ = 2.8–27.6°
*b* = 5.685 (2) Å	µ = 1.76 mm^−^^1^
*c* = 7.894 (3) Å	*T* = 298 K
β = 93.864 (18)°	Block, brown
*V* = 706.3 (5) Å^3^	0.18 × 0.17 × 0.17 mm
*Z* = 2	

### Data collection

**Table:**

Bruker SMART CCD area-detector diffractometer	1532 independent reflections
Radiation source: fine-focus sealed tube	1296 reflections with *I* > 2σ(*I*)
graphite	*R*_int_ = 0.029
ω scans	θ_max_ = 27.0°, θ_min_ = 2.6°
Absorption correction: multi-scan (*SADABS*; Sheldrick, 1996)	*h* = −20→18
*T*_min_ = 0.743, *T*_max_ = 0.755	*k* = −7→7
4102 measured reflections	*l* = −10→9

### Refinement

**Table:**

Refinement on *F*^2^	Primary atom site location: structure-invariant direct methods
Least-squares matrix: full	Secondary atom site location: difference Fourier map
*R*[*F*^2^ > 2σ(*F*^2^)] = 0.030	Hydrogen site location: inferred from neighbouring sites
*wR*(*F*^2^) = 0.084	H atoms treated by a mixture of independent and constrained refinement
*S* = 1.04	*w* = 1/[σ^2^(*F*_o_^2^) + (0.0495*P*)^2^ + 0.0905*P*] where *P* = (*F*_o_^2^ + 2*F*_c_^2^)/3
1532 reflections	(Δ/σ)_max_ = 0.001
100 parameters	Δρ_max_ = 0.45 e Å^−^^3^
1 restraint	Δρ_min_ = −0.30 e Å^−^^3^

### Special details

**Table:**

Geometry. All e.s.d.'s (except the e.s.d. in the dihedral angle between two l.s. planes) are estimated using the full covariance matrix. The cell e.s.d.'s are taken into account individually in the estimation of e.s.d.'s in distances, angles and torsion angles; correlations between e.s.d.'s in cell parameters are only used when they are defined by crystal symmetry. An approximate (isotropic) treatment of cell e.s.d.'s is used for estimating e.s.d.'s involving l.s. planes.
Refinement. Refinement of *F*^2^ against ALL reflections. The weighted *R*-factor *wR* and goodness of fit *S* are based on *F*^2^, conventional *R*-factors *R* are based on *F*, with *F* set to zero for negative *F*^2^. The threshold expression of *F*^2^ > σ(*F*^2^) is used only for calculating *R*-factors(gt) *etc*. and is not relevant to the choice of reflections for refinement. *R*-factors based on *F*^2^ are statistically about twice as large as those based on *F*, and *R*- factors based on ALL data will be even larger.

### Fractional atomic coordinates and isotropic or equivalent isotropic displacement parameters (Å^2^)

**Table:**

	*x*	*y*	*z*	*U*_iso_*/*U*_eq_	
Ni1	0.5000	0.5000	0.0000	0.03469 (15)	
Cl1	0.06466 (4)	0.34882 (15)	0.19374 (11)	0.0778 (3)	
N1	0.45858 (11)	0.2416 (3)	0.1094 (2)	0.0421 (4)	
O1	0.39683 (9)	0.6527 (2)	−0.01584 (18)	0.0411 (3)	
C1	0.31247 (13)	0.3565 (3)	0.1140 (3)	0.0374 (4)	
C2	0.32363 (13)	0.5745 (4)	0.0315 (3)	0.0369 (4)	
C3	0.25045 (14)	0.7137 (4)	−0.0045 (3)	0.0445 (5)	
H3	0.2554	0.8554	−0.0619	0.053*	
C4	0.17234 (15)	0.6450 (4)	0.0433 (3)	0.0501 (6)	
H4	0.1252	0.7403	0.0189	0.060*	
C5	0.16322 (14)	0.4331 (5)	0.1283 (3)	0.0493 (6)	
C6	0.23191 (14)	0.2906 (4)	0.1628 (3)	0.0439 (5)	
H6	0.2252	0.1487	0.2190	0.053*	
C7	0.38261 (14)	0.1990 (4)	0.1480 (3)	0.0412 (5)	
H7	0.3722	0.0571	0.2015	0.049*	
H1	0.4993 (14)	0.133 (4)	0.131 (4)	0.080*	

### Atomic displacement parameters (Å^2^)

**Table:**

	*U*^11^	*U*^22^	*U*^33^	*U*^12^	*U*^13^	*U*^23^
Ni1	0.0430 (2)	0.0274 (2)	0.0335 (2)	0.00107 (14)	0.00128 (15)	0.00308 (13)
Cl1	0.0488 (4)	0.0872 (6)	0.0994 (6)	−0.0016 (3)	0.0197 (3)	0.0169 (4)
N1	0.0470 (10)	0.0325 (9)	0.0465 (10)	0.0054 (7)	0.0023 (8)	0.0076 (7)
O1	0.0437 (8)	0.0314 (7)	0.0483 (9)	0.0025 (6)	0.0049 (6)	0.0063 (6)
C1	0.0467 (11)	0.0305 (10)	0.0348 (10)	−0.0012 (8)	0.0020 (8)	−0.0010 (8)
C2	0.0447 (11)	0.0312 (9)	0.0345 (10)	0.0018 (8)	0.0017 (8)	−0.0021 (8)
C3	0.0510 (12)	0.0351 (11)	0.0469 (13)	0.0042 (9)	0.0004 (10)	0.0025 (9)
C4	0.0470 (12)	0.0463 (14)	0.0565 (14)	0.0086 (10)	−0.0006 (10)	−0.0021 (10)
C5	0.0454 (13)	0.0497 (13)	0.0530 (14)	−0.0026 (10)	0.0052 (10)	−0.0009 (11)
C6	0.0517 (12)	0.0379 (11)	0.0424 (12)	−0.0045 (10)	0.0060 (9)	0.0008 (9)
C7	0.0513 (12)	0.0316 (10)	0.0406 (11)	−0.0007 (9)	0.0037 (9)	0.0066 (8)

### Geometric parameters (Å, °)

**Table:**

Ni1—O1^i^	1.8414 (15)	C1—C7	1.434 (3)
Ni1—O1	1.8414 (15)	C2—C3	1.412 (3)
Ni1—N1^i^	1.8455 (18)	C3—C4	1.370 (3)
Ni1—N1	1.8455 (18)	C3—H3	0.93
Cl1—C5	1.738 (2)	C4—C5	1.391 (4)
N1—C7	1.280 (3)	C4—H4	0.93
N1—H1	0.897 (10)	C5—C6	1.365 (3)
O1—C2	1.315 (2)	C6—H6	0.93
C1—C6	1.403 (3)	C7—H7	0.93
C1—C2	1.417 (3)		
			
O1^i^—Ni1—O1	180.00 (4)	C4—C3—C2	121.5 (2)
O1^i^—Ni1—N1^i^	93.89 (7)	C4—C3—H3	119.2
O1—Ni1—N1^i^	86.11 (7)	C2—C3—H3	119.2
O1^i^—Ni1—N1	86.11 (7)	C3—C4—C5	120.3 (2)
O1—Ni1—N1	93.89 (7)	C3—C4—H4	119.9
N1^i^—Ni1—N1	180.00 (10)	C5—C4—H4	119.9
C7—N1—Ni1	128.97 (15)	C6—C5—C4	120.2 (2)
C7—N1—H1	120 (2)	C6—C5—Cl1	119.4 (2)
Ni1—N1—H1	111 (2)	C4—C5—Cl1	120.37 (19)
C2—O1—Ni1	127.63 (13)	C5—C6—C1	120.6 (2)
C6—C1—C2	120.15 (18)	C5—C6—H6	119.7
C6—C1—C7	118.94 (19)	C1—C6—H6	119.7
C2—C1—C7	120.91 (19)	N1—C7—C1	124.10 (19)
O1—C2—C3	118.37 (19)	N1—C7—H7	118.0
O1—C2—C1	124.41 (18)	C1—C7—H7	118.0
C3—C2—C1	117.21 (19)		

Symmetry codes: (i) −*x*+1, −*y*+1, −*z*.

### Hydrogen-bond geometry (Å, °)

**Table:**

*D*—H···*A*	*D*—H	H···*A*	*D*···*A*	*D*—H···*A*
C7—H7···O1^ii^	0.93	2.54	3.318 (3)	142

Symmetry codes: (ii) *x*, −*y*+1/2, *z*+1/2.
